# Safety of Human Papillomavirus 9-Valent Vaccine: A Meta-Analysis of Randomized Trials

**DOI:** 10.1155/2017/3736201

**Published:** 2017-07-24

**Authors:** Ana Paula Ferreira Costa, Ricardo Ney Oliveira Cobucci, Janine Medeiros da Silva, Paulo Henrique da Costa Lima, Paulo César Giraldo, Ana Katherine Gonçalves

**Affiliations:** ^1^Postgraduate Program in Health Sciences, Federal University of Rio Grande do Norte, Natal, RN, Brazil; ^2^Department of Gynecology and Obstetrics, University Potiguar (UnP), Natal, RN, Brazil; ^3^Department of Gynecology and Obstetrics, Federal University of Rio Grande do Norte (UFRN), Natal, RN, Brazil; ^4^Department of Gynecology and Obstetrics, State University of Campinas (UNICAMP), Campinas, SP, Brazil

## Abstract

Vaccination against human papillomavirus (HPV) has been progressively implemented in most developed countries for approximately 10 years. In order to increase the protection of the vaccines, a 9-valent vaccine (HPV9) was developed, which provides protection against nine types of the virus. Studies evaluating its safety are rare. Thus, we performed a meta-analysis of three clinical trials assessing adverse effects on women randomly vaccinated with HPV9 or tetravalent vaccine (HPV4), with the objective of analyzing whether the HPV9 is as safe as HPV4. An electronic data search was performed through the PubMed, Embase, Scopus, Web of Science, and SciELO databases. The studies selected 27,465 women who received one of the two vaccines. Pain (OR 1.72; 95% CI 1.62–1.82) and erythema (OR 1.29; 95% CI 1.21–1.36) occurred significantly more in the HPV9 group. However, there was no significant difference between the groups for the following adverse effects: headache (OR 1.07; 95% CI 0.99–1.15), dizziness (OR 1.09; 95% CI 0.93–1.27), and fatigue (OR 1.09; 95% CI 0.91–1.30), and the occurrence of serious events related to vaccination was similarly rare among those vaccinated. Therefore, our findings demonstrate that HPV9 in female patients is as safe as the tetravalent vaccine.

## 1. Introduction

The human papillomavirus (HPV) can cause cervical premalignant and malignant lesions [1, 2] as well as genital warts [3, 4]. Vaccines directed against the most relevant HPV types have collaborated to prevent virus-related diseases [5]. Three effective vaccines are approved by the Food and Drug Administration (FDA): the bivalent vaccine (HPV2), which protects against HPV types 16 and 18; the tetravalent vaccine (HPV4), which protects against types 16, 18, 6, and 11; the 9-valent vaccine (HPV9), which protects against types 6, 11, 16, 18, 31, 33, 45, 52, and 58 [6, 7].

The effects of HPV vaccination programs on population health have already been observed in the form of reduced incidences of HPV infections, genital warts, and HPV-attributed precancerous lesions. However, it is too early to study the effects of vaccination on cervical cancer rates, as it takes decades for HPV infection to progress to cervical cancer [8].

Since the vaccination programs started, several safety and efficacy surveillance protocols for the vaccines have been implemented [9]. Some are passive, such as the Vaccine Adverse Event Reporting System (VAERS) in the United States, which showed that the postvaccination adverse event rates with the HPV4 were not higher than the historical rates of other vaccines [10]. Others use more active surveillance such as the multicenter study in seven health care organizations in the United States, on women between 9 and 26 years, who received 600,558 doses of HPV4; the purpose of which was to monitor certain adverse events, such as Guillain-Barre Syndrome, cerebrovascular accident, venous thromboembolism, appendicitis, convulsions, syncope, allergic reactions, and anaphylaxis. No meaningful increase was found in the risk of predetermined objectives [11].

The fact that HPV vaccination showed positive outcomes in several countries has contributed to the development of HPV9 to increase protection against five more strains (i.e., HPV types 31, 33, 45, 52, and 58), making it nine HPV strains. Such a vaccine has the potential to offer protection against approximately 90% of cervical cancers, an increase from the 70% offered by the HPV4. HPV9 is similar in composition to the tetravalent vaccine, using virus-like particles to elicit immune responses [8].

Recipients of the 9-valent vaccine were slightly more likely to experience adverse events than recipients of the tetravalent vaccine were, possibly owing to the higher amounts of virus-like particles and adjuvants in the HPV9 [8]. This meta-analysis aims to assess whether HPV9 is as safe as HPV4 in the female population.

## 2. Materials and Methods

### 2.1. Study Design

This meta-analysis follows the Preferred Reporting Items for Systematic Reviews and Meta-Analyses (PRISMA) guidelines [12].

Two researchers (APFC and JMS) performed the selection of the studies of interest. Subsequently, data were extracted by three other researchers (APFC, AKS, and RNC) according to the data extraction protocol. They evaluated the studies found based on the following inclusion criteria: (1) randomized controlled trial- (RCT-) type studies that evaluated the side effects of HPV4, Gardasil, and HPV9 (Gardasil9); (2) experiments involving women; (3) studies evaluating the safety, immunogenicity, and efficacy parameters of the vaccines; and (4) studies that presented similar vaccination protocols. The exclusion criteria were as follows: (1) studies involving men, (2) pregnant women, (3) women who were vaccinated only with 9-valent vaccine, and (4) observational studies. All discrepancies between these three reviewers were resolved by the consensus of all authors.

### 2.2. Search Strategy

**Figure 1:**
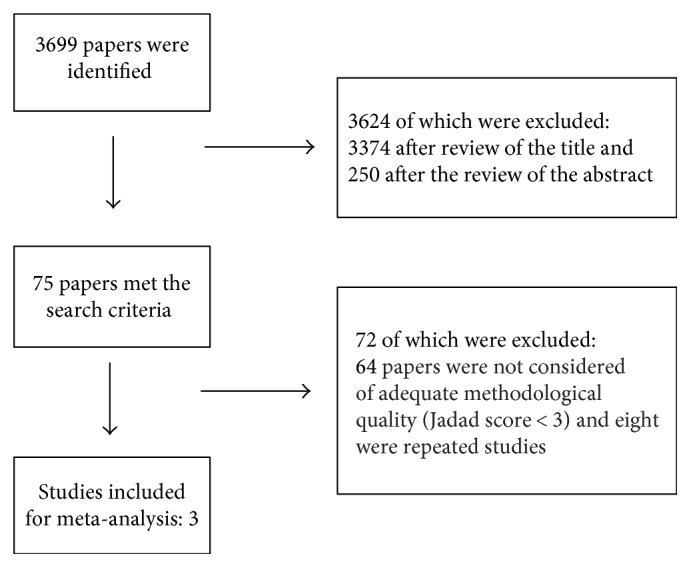
Flow diagram of the selection process of studies.

The research was performed by a wide and comprehensive search of literature from databases (PubMed, Embase, Web of Science, Scopus, and SciELO) until December 2016. The following descriptors were used: (HPV OR Human papillomavirus) AND (vaccines OR vaccination) OR (tetravalent HPV vaccine) OR (9-valent vaccine) AND (side effects) OR (adverse events) AND (randomized controlled trial) OR (double blind method) OR (clinical trial). No language restrictions were applied. The flowchart of this study is shown in Figure 1.

### 2.3. Data Analysis

Data were entered in the Review Manager software (RevMan 5.2), which allows the user to enter protocols as well as complete reviews, including text, features of studies, comparison tables, and study data, as well as to perform meta-analysis of the entered data.

To evaluate the safety and efficacy between the 9-valent and tetravalent vaccines, dichotomous data were extracted from each study and were inserted into a 2 × 2 contingency table, with subsequent individual determination of odds ratio (OR), to obtain a summarized overall estimate. Fixed-effects or random-effects models were chosen depending on whether there was an absence or presence of heterogeneity between studies. Heterogeneity was assessed by the I2 statistic. Statistical heterogeneity between studies was assessed by the I2 statistic (<25%, no heterogeneity; 25%–50%, moderate heterogeneity; and >50%, strong heterogeneity). When a significant heterogeneity existed across the included studies (I2 > 50%), a random-effects model was used for the analysis; otherwise, the fixed-effects model was used [13, 14]. In addition, we use the Egger funnel plot to assess possible publication bias [13].

A Jadad score, which was based on the following three subscales: randomization (2–0), blind (2–0), and withdrawals and dropouts (1–0), assessed the study quality. For every answer of yes, unclear, or not, the values of 2 to 0 points were assigned, respectively. In our analysis, we judged that the studies evaluated that had a score ≥ 3 would be considered high quality. The level of evidence of each study (Table 1) was defined according to the definitions of the Oxford Centre for Evidence-Based Medicine [15].

We chose to use the fixed-effects model during statistical analysis, because this model is applied when there is little variability between the results of studies [16]. To determine OR, we used a confidence interval (CI) of 95% with values of *P* < 0.05 considered statistically significant.

## 3. Results

Three studies were included for meta-analysis involving 27,465 women who received the HPV9 or HPV4 [6, 17, 18] (Table 1).

**Figure 2:**
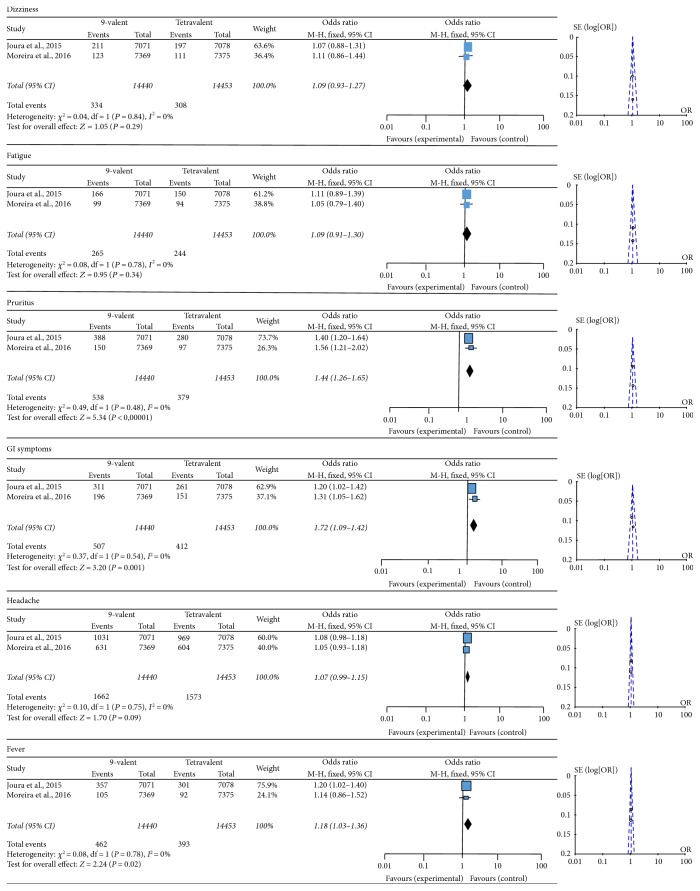
Forest and funnel plots of systemic adverse effects.

Rates of systemic events such as headaches (OR 1.07; 95% CI 0.99–1.15), dizziness (OR 1.09; 95% CI 0.93–1.27), and fatigue (OR 1.09; 95% CI 0.91–1.30) were similar between 9-valent and tetravalent vaccine groups (Figure 2). However, women vaccinated with HPV9 presented more fever (OR 1.18; 95% CI 1.03–1.36), pruritus (OR 1.44; 95% CI 1.26–1.15), and gastrointestinal (GI) symptoms: diarrhea, nausea, and vomiting (OR 1.24; 95% CI 1.09–1.45) (Figure 2).

**Figure 3:**
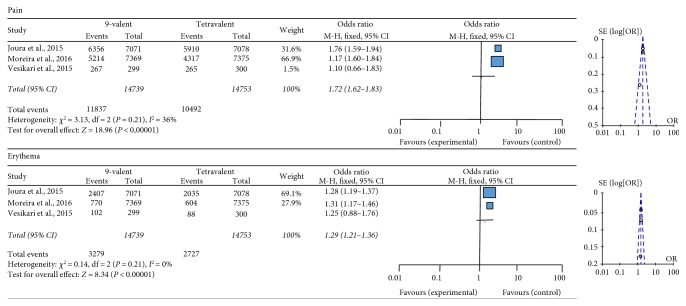
Forest and funnel plots of injection site-related adverse effects.

Injection site-related adverse effects like pain (OR 1.72; 95% CI 1.62–1.82) and erythema (OR 1.29; 95% CI 1.21–1.36) occurred significantly more in the HPV9 group (Figure 3).

Out of more than 27,000 vaccine recipients, a total of 29 and 23 recipients from the HPV9 and HPV4 groups, respectively, experienced a serious vaccine-related adverse event. A total of 6 deaths were recorded from each group but none was judged to be vaccine related.

## 4. Discussion

In clinical trials and meta-analysis, the tetravalent HPV vaccine was found to be safe and efficacious [8, 9]. A recent observational study with human papillomavirus 9-valent vaccine shows that administration of the HPV9 was generally well tolerated. A lower proportion of girls (81.9%) and boys (72.8%) compared to that of young women (85.4%) reported injection-site adverse effects; most of which were mild to moderate in intensity [19].

In this meta-analysis, occurrence of adverse effects was reported in all RCTs [6, 17, 18]. The most reported side effects (SE) were injection-site reactions; most of these SE were mild or moderate in intensity. The most common among subjects who received the HPV9 vaccine when compared with HPV4 subjects were pain and erythema, seen in approximately 80% and 22%, respectively. Headache, fever, pruritus, and GI symptoms were the most common vaccine-related systemic SE of all participants; however, just fever, pruritus, and GI symptoms were significantly more reported in women vaccinated with HPV9. The 9-valent vaccine recipients were slightly more likely to experience these adverse events than tetravalent vaccine recipients were, and this possibly occurs due to the higher amounts of virus-like particles and adjuvants in the 9-valent vaccine, as well as serotypes [8].

Nowadays, in the scientific literature, only observational studies on the adverse effects of the 9-valent vaccine, without comparison with the side effects of the tetravalent vaccine, were found. Thus, this innovative study was compiled in a meta-analysis of randomized clinical trials, involving only women aged 9–26 years, vaccinated by HPV9 or by the tetravalent vaccine, by comparing adverse effects between the two groups.

Serious vaccine-related adverse events and deaths were not common, and there was no significant difference in both groups in our analysis. Furthermore, no vaccine-related deaths were reported. Some observational studies also found similarly low levels of vaccine-related severe adverse events and no vaccine-related deaths [19–22].

This meta-analysis was conducted using only the RCT, thus greatly reducing the possibility of bias. Since the authors of the different trials used the same vaccination protocols, confounding bias was therefore minimized. Publication bias is not believed to have occurred as shown by the funnel plots. However, the fact that Joura et al.'s study selected only women between 16 and 26 years old establishes a bias and prevents our results from being considered in the female population aged between 11 and 15 years, which is included in the current vaccination recommendations for both vaccines [6].

The results of our study should be interpreted with some caution because it has limitations. First, the work has been conducted using three RCTs only, and the selected studies investigated just women with different age ranges and had different sample sizes. The short follow-up period of the selected studies is also responsible for other potential weaknesses of the data.

## 5. Conclusions

Despite the limitations discussed above, the results of our meta-analysis show that the 9-valent vaccine in female patients is as safe as the tetravalent vaccine. However, firm results to generalize our findings among specific populations, such as men, are precluded by the small number of enrolled studies involving only women. Thus, future research in the field becomes necessary.

## Figures and Tables

**Table 1:** Description of the characteristics of included studies.

Author, year	Country	Design of study	Jadad	Follow-up	Age range (y)	Sample size
Joura et al., 2015	Asia-Pacific, Europe, Latin America, and North America	RCT	5	7 months	16–26	14,215
Vesikari et al., 2015	Belgium, Denmark, Finland, Italy, Spain, and Sweden	RCT	5	7 months	9–15	600
Moreira et al., 2016	Africa, Asia-Pacific, Europe, Latin America, and North America	RCT	5	7 months	9–26	12,650

RCT: randomized controlled trial.
